# Supplementary material for: A control theory approach to optimal pandemic mitigation
Source: PLoS One. 2021 Feb 19;16(2):e0247445. doi: 10.1371/journal.pone.0247445 (PMC7894916; doi:10.1371/journal.pone.0247445)
Supplement: S1 Appendix — 1) First order necessary conditions for optimality. 2) Stability analysis of the uncontrolled SIR model with finite immune response. (PDF) [file pone.0247445.s001.pdf]

S1 Appendix

A control theory approach to optimal pandemic mitigation

Prakhar Godara[1], Stephan Herminghaus[1], Knut M. Heidemann [1*]
1 Max Planck Institute for Dynamics and Self-Organization, Am Fassberg 17, 37073 Göttingen, Germany

* knut.heidemann@ds.mpg.de

First order necessary conditions for optimality

The problem of optimal control is given in Eq. (8) in the main manuscript. Here we derive the necessary conditions for optimality, Eqs. (10)-(13), from the main manuscript. Defining $\psi(\boldsymbol{x}(t)) = I(t) - I_h$ we rewrite the functional in Eq. (9):

$$J\{\alpha\} = \int_0^{t_e} f(\alpha(t)) + \boldsymbol{\lambda}(t) \cdot [\dot{\boldsymbol{x}}(t) - \boldsymbol{h}(\boldsymbol{x}, \alpha(t))] + \mu(t)\psi(\boldsymbol{x}(t))\, dt\,, \tag{1}$$

with the complimentary slackness condition $\mu(t)\psi(\boldsymbol{x}^*(t)) = 0$ and $\mu(t) \geq 0$. The slackness condition can be seen as activation of the constraint, i.e., in the region when the optimal trajectory satisfies $\psi(\boldsymbol{x}^*(t)) < 0$ the Lagrange multiplier is 0, or in other words the constraint is inactive.

The first order conditions for optimality can be found by setting the first variation of the functional $\delta J = 0$. To this end we consider the variations $\boldsymbol{x}(t) = \boldsymbol{x}^*(t) + \eta\,\boldsymbol{\sigma}(t)$, $\alpha(t) = \alpha^*(t) + \eta\,\theta(t)$ and $t_e = t_e^* + \eta\,\chi$ for some infinitesimal scalar parameter η. Upon making the substitutions we have:

$$\begin{aligned} J = J^* + \eta\,\delta J = \int_0^{t_e^*+\eta\chi} & f(\alpha^*(t)) + \partial_\alpha f|_{\alpha^*(t)}\eta\,\theta(t) + \boldsymbol{\lambda}(t) \cdot \big[\dot{\boldsymbol{x}}^*(t) + \eta\,\dot{\boldsymbol{\sigma}}(t) - \boldsymbol{h}(\boldsymbol{x}^*(t), \alpha^*(t)) - \\ & \boldsymbol{\nabla}_{\boldsymbol{x}}\boldsymbol{h}|_{\boldsymbol{x}^*(t)} \cdot \eta\,\boldsymbol{\sigma}(t) - \partial_\alpha \boldsymbol{h}|_{\alpha^*(t)} \cdot \eta\,\theta(t)\big] + \mu(t)\psi(\boldsymbol{x}^*(t)) + \boldsymbol{\nabla}_{\boldsymbol{x}}\psi|_{\boldsymbol{x}^*(t)} \cdot \eta\,\boldsymbol{\sigma}(t)\mu(t)\, dt\,, \end{aligned} \tag{2}$$

where $J^* = \int_0^{t_e^*} f(\alpha^*(t)) + \boldsymbol{\lambda}(t) \cdot [\dot{\boldsymbol{x}}^*(t) - \boldsymbol{h}(\boldsymbol{x}^*(t), \alpha^*(t))] + \mu(t)\psi(\boldsymbol{x}^*(t))\, dt$. Now noting that $\int_0^{t_e^*+\eta\chi} A(t)\, dt = \int_0^{t_e^*} A(t)\, dt + \int_{t_e^*}^{t_e^*+\eta\chi} A(t)\, dt$ and $\int_{t_e^*}^{t_e^*+\eta\chi} A(t)\, dt = A(t_e^*)\eta\chi$, and considering terms only up to first order in η, we have

$$\begin{aligned} \delta J = & \Big[f(\alpha^*(t_e^*)) + \boldsymbol{\lambda}(t_e^*) \cdot [\dot{\boldsymbol{x}}(t_e^*) - \boldsymbol{h}(\boldsymbol{x}(t_e^*), \alpha^*(t_e^*))] + \mu(t_e^*)\psi(\boldsymbol{x}^*(t_e^*))\Big]\chi \\ & + \int_0^{t_e^*} \partial_\alpha f|_{\alpha^*(t)}\theta(t) + \boldsymbol{\lambda}(t) \cdot \big[\dot{\boldsymbol{\sigma}}(t) - \boldsymbol{\nabla}_{\boldsymbol{x}}\boldsymbol{h}|_{\boldsymbol{x}^*(t)} \cdot \boldsymbol{\sigma}(t) - \partial_\alpha \boldsymbol{h}|_{\alpha^*(t)}\theta(t)\big] + \boldsymbol{\nabla}_{\boldsymbol{x}}\psi|_{\boldsymbol{x}^*(t)} \cdot \boldsymbol{\sigma}(t)\mu(t)\, dt\,. \end{aligned} \tag{3}$$

Making use of the slackness condition, performing integration by parts on the $\boldsymbol{\lambda}(t) \cdot \dot{\boldsymbol{\sigma}}(t)$ term, and rearranging the terms we get:

$$\delta J = \Big[f(\alpha^*(t_e^*)) + \boldsymbol{\lambda}(t_e^*) \cdot [\dot{\boldsymbol{x}}^*(t_e^*) - \boldsymbol{h}(\boldsymbol{x}^*(t_e^*), \alpha^*(t_e^*))] \Big] \chi + \int_0^{t_e^*} \theta(t) \Big[\partial_\alpha f|_{\alpha^*(t)} - \boldsymbol{\lambda}(t) \cdot \partial_\alpha \boldsymbol{h}|_{\alpha^*(t)} \Big] dt$$
$$+ \int_0^{t_e^*} \boldsymbol{\sigma}(t) \cdot \Big[- \dot{\boldsymbol{\lambda}}(t) - \boldsymbol{\lambda}(t) \cdot \boldsymbol{\nabla}_{\boldsymbol{x}} \boldsymbol{h}|_{\boldsymbol{x}^*(t)} + \boldsymbol{\nabla}_{\boldsymbol{x}} \psi|_{\boldsymbol{x}^*(t)} \mu(t) \Big] dt + [\boldsymbol{\lambda}(t) \cdot \boldsymbol{\sigma}(t)]_0^{t_e^*} . \tag{4}$$

For the last term we make use of the initial conditions $\boldsymbol{x}(0) = \boldsymbol{x}_0$, hence we require $\boldsymbol{\sigma}(0) = \mathbf{0}$. The terminal constraint implies $R_0^{-1} = S(t_e) = S(t_e^* + \eta\chi) = S^*(t_e^*) + \eta(\dot{S}^*(t_e^*)\chi + \sigma_s(t_e^*)) + \mathcal{O}(\eta^2)$, and therefore $\dot{S}^*(t_e^*)\chi + \sigma_s(t_e^*) = 0$. Taking these into account we finally get the first order necessary conditions for optimal control:

$$f(\alpha^*(t_e^*)) - \boldsymbol{\lambda}(t_e^*) \cdot \boldsymbol{h}(\boldsymbol{x}^*(t_e^*), \alpha^*(t_e^*)) = 0 \,, \tag{5}$$
$$\dot{\boldsymbol{\lambda}}(t) = -\boldsymbol{\lambda}(t) \cdot \boldsymbol{\nabla}_{\boldsymbol{x}} \boldsymbol{h}|_{\boldsymbol{x}^*(t)} + \boldsymbol{\nabla}_{\boldsymbol{x}} \psi|_{\boldsymbol{x}^*(t)} \mu(t) \,, \tag{6}$$
$$\partial_\alpha f|_{\alpha^*(t)} - \boldsymbol{\lambda}(t) \cdot \partial_\alpha \boldsymbol{h}|_{\alpha^*(t)} = 0 \,, \tag{7}$$
$$\lambda_I(t_e^*) = 0 \,. \tag{8}$$

Stability analysis of the uncontrolled SIR model with finite immune response

The uncontrolled SIR model with finite immune response (Eq. 24 in the main manuscript with $\alpha = 0$) can be written as:

$$\begin{aligned} \partial_t S &= -\tfrac{1}{\tau} R_0 S I + \tfrac{1}{\rho} \left(1 - S - I\right) , \\ \partial_t I &= \tfrac{1}{\tau} (R_0 S I - I) \,. \end{aligned} \tag{9}$$

It has a fixed point at

$$x_\infty = (S_\infty, I_\infty) = \left(\frac{1}{R_0}, \frac{1 - 1/R_0}{1 + \rho/\tau} \right) . \tag{10}$$

Linear stability analysis requires computation of the Jacobian of the dynamical system evaluated at the fixed point. It is given via:

$$J|_{x_\infty} = \begin{bmatrix} \frac{1-R_0}{\tau+\rho} - 1/\rho & -1/\tau - 1/\rho \\ \frac{R_0 - 1}{\tau+\rho} & 0 \end{bmatrix} . \tag{11}$$

Its eigenvalues are given by:

$$\lambda_{1,2} = -\frac{\rho R_0 + \tau}{2\rho(\tau+\rho)} \pm \sqrt{\left(\frac{\rho R_0 + \tau}{2\rho(\tau+\rho)} \right) + \frac{1 - R_0}{\tau\rho}} \,. \tag{12}$$

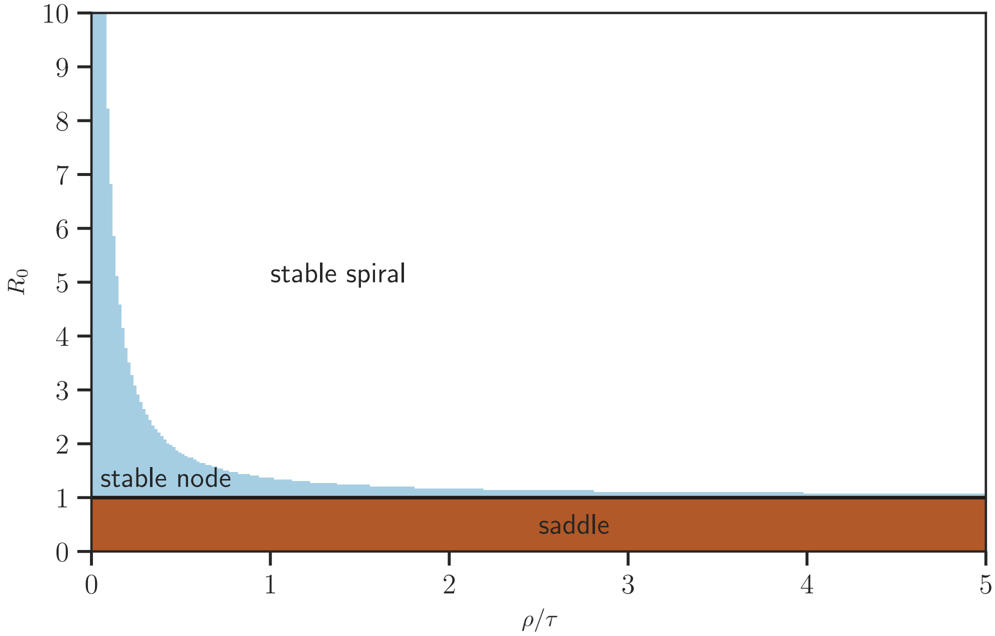

Fig 1. Stability of the fixed point x_∞ of the uncontrolled ($\alpha = 0$) SIR model with finite immune response (Eq. 9) for different values of R_0 and ρ/τ. The color encodes the three different regimes.

The first term is always negative, we thus have three regimes:

$$\begin{aligned} \lambda_{1,2} \in \mathbb{R} \ \wedge \ \lambda_{1,2} < 0 : & \quad \text{stable node} \\ \lambda_{1,2} \in \mathbb{R} \ \wedge \ \lambda_1 > 0,\ \lambda_2 < 0 : & \quad \text{saddle} \\ \mathrm{Im}(\lambda_{1,2}) \neq 0 \ \wedge \ \mathrm{Re}(\lambda_{1,2}) < 0 : & \quad \text{stable spiral} \end{aligned} \tag{13}$$

In Fig. S1 we show the different regimes of stability. The fixed point x_∞ is stable for any $R_0 \geq 1$. The *stable node* regime is limited to a very small, rather unrealistic parameter regime (mainly $\rho \leq \tau$, i.e., loss of immunity is faster than recovery from the infection). So for most cases, the fixed point is a *stable spiral*, in particular for the estimated values for SARS-CoV-2 ($\rho/\tau \approx 93$, $R_0 \approx 3$).
