# Supplementary material for: A control theory approach to optimal pandemic mitigation
Source: PLoS One. 2021 Feb 19;16(2):e0247445. doi: 10.1371/journal.pone.0247445 (PMC7894916; doi:10.1371/journal.pone.0247445)
Supplement: Attachment — Submitted filename: response-to-reviewers.pdf [file pone.0247445.s002.pdf]

Rebuttal Letter

For the manuscript ”A control theory approach to optimal pandemic mitigation“ [PONE-D-20-30011]

January 28, 2021

Response to the Editor

1. Please ensure that your manuscript meets PLOS ONE’s style requirements, including those for file naming.

   `https://journals.plos.org/plosone/s/file?id=wjVg/PLOSOne_formatting_sample_main_body.pdf` and

   `https://journals.plos.org/plosone/s/file?id=ba62/PLOSOne_formatting_sample_title_authors_affiliations.pdf`

2. *Thank you for stating the following in the Acknowledgments Section of your manuscript:*

   ”P. G., S. H., and K. M. H. gratefully acknowledge support from the Max Planck Society.”

   We note that you have provided funding information that is not currently declared in your Funding Statement. However, funding information should not appear in the Acknowledgments section or other areas of your manuscript. We will only publish funding information present in the Funding Statement section of the online submission form.

   a) *Please remove any funding-related text from the manuscript and let us know how you would like to update your Funding Statement. Currently, your Funding Statement reads as follows:*

      ”The author(s) received no specific funding for this work.”

      Response: We removed the funding-related text from the manuscript, as requested.

      —

   b) Please include your amended statements within your cover letter; we will change the online submission form on your behalf.

Response: Our funding statement should read:

”P. G., S. H., and K. M. H. gratefully acknowledge support from the Max Planck Society.”

Reviewer 1

We highly appreciate your comments regarding our manuscript. We section our response by addressing your specific remarks:

1. *Figures are of –very– low quality (resolution). Figure captions are highly insufficient. For example, in Fig. 1 the cost function f(alpha) is mentioned as 'defined somewhere in the text'. Suggesting that the reader should take the trouble to search for her/him-self. That is arrogant.*

   Response: Thank you for pointing out that the figures that you received are of poor resolution. We submitted high resoltion figures, hence we will check with the production team of PLOS ONE to figure out what happened and make sure that the final figures appearing in the publication will be of good quality.

   We checked all figure captions for clarity and completeness (see the marked changes in the revised manuscript).

   Regarding your specific comment on Fig. 1. In our manuscript we write:

   > "The dashed and dotted curves are possible choices for mitigation measures; such curves correspond to the *cost functions* referred to in the manuscript."

   You are right, this is indeed unclear. We added a reference to Eq. 7, which defines the term cost function in mathematical sense.

   —

2. *Herd immunity is taken at the endpoint. In reality, the outbreak will take substantially longer, unit I(t)=0. There are not estimates about the costs incurring after the herd immunity point. Without this estimate, the paper cannot be published.*

   Response: Thank you for this remark. In the paper, we only consider costs incurred due to mitigation measures. Hence, there are no addtional costs after herd immunity has been reached.

   Indeed, if we were to introduce additional costs due to the number of infections (e.g., fatalities), we would accumulate costs even after herd immunity has been reached. We take the costs associated with infections into account implicitly by introducing the hospital capacity I_h, which limits the number of simultaneous infections in order to prevent large case fatality rates.

   We refrain from defining separate cost terms for both mitigation measures and number of infected individuals to 1) keep the model mathematically minimal, allowing for an analytical treatment, and 2) avoid the difficulty of weighting societal costs due to mitigation measures against costs due to infections. Moreover, since we aim for reaching herd immunity and vaccination is not available, there is no

way around a fraction of $1 - 1/R_0$ of the population going through the infection. We added a corresponding paragraph to the conclusions of the manuscript.

—

3. *The incurring costs are taken to instantaneous. In reality, a certain level of containment may be ok for a few weeks, but disastrous if uphold for many years, as suggested by the authors. For example, for a few weeks/month business may close and reopen, but not after many years. The authors should clearly state that they propose a duration of 4-6 years (you need to include the post-here period!) for Germany. The issue of additional costs incurring for prolong lockdowns need to be discussed.*

   Response: Thank you, this is indeed an important remark. Prolonged mitigation measures (e.g., lockdowns) may induce disproportionally higher costs. Indeed, one could consider cost functions being nonlocal in time, i.e., where costs incurred at time t depend on the history of the system (Have there been strong measures before?). This would be a highly relevant and interesting extension; it would go beyond the complexity of our paper, though. We added a discussion on this point in the manuscript.

   We present the *end of mitigation measures* as duration of the problem because this is the period were costs are beeing incurred. In the remaining time, the number of infections will stay below HSS capacity I_h, without any measures being applied. This period, after herd immunity, is in principle infinite, since for permanent immunity ($\rho = \infty$), $I(t) = 0$ only for $t \to \infty$. For finite immune response, the number of infected converges to a steady state $I_\infty = (1 - 1/R_0)/(1 + \rho/\tau)$, which is greater than zero for any finite ρ. Hence it is not possible to define the duration until $I(t) = 0$ in a consistent way.

Reviewer 2

Comments to the authors: *This manuscript introduce a control theory approach to identify optimal pandemic mitigation strategies based on the framework of homogeneous susceptible-infected-recovered (SIR) models. It provides an optimal control strategy. Optimality is derived and verified by variational and numerical methods for a number of model cost functions. And also they discussed in terms of the feasibility of strategies based on herd immunity. It has certain theoretical guiding significance.*

Response: We appreciate your positive feedback concerning our manuscript.

Further major changes in the manuscript

All changes in the manuscript are highlighted. In addition to the revisions based on the reviewers' remarks, there are two major changes (see below):

1. We updated the estimate for the average duration of infection to $\tau = 10$ days (before: 14 days).
2. We added phase III (no mitigation measures) to Fig. 6 and adjusted the paragraph referring to it.
